# Magnetic Graphene Oxide: Effect of Preparation Route on Reactive Black 5 Adsorption

**DOI:** 10.3390/ma6041360

**Published:** 2013-03-28

**Authors:** George Z. Kyzas, Nikolina A. Travlou, Orestis Kalogirou, Eleni A. Deliyanni

**Affiliations:** 1 Department of Petroleum and Natural Gas Technology, Technological Educational Institute of Kavala, Kavala GR–654 04, Greece; 2 Department of Chemistry, Aristotle University of Thessaloniki, Thessaloniki GR–541 24, Greece; E-Mails: nikolinatrav@gmail.com (N.A.T.); lenadj@chem.auth.gr (E.A.D.); 3 Department of Physics, Aristotle University of Thessaloniki, Thessaloniki GR–541 24, Greece; E-Mail: orestis.kalogirou@physics.auth.gr

**Keywords:** magnetic graphene oxide, preparation, impregnation, co-precipitation, adsorption, reactive dye

## Abstract

In this study, the effect of preparation route of magnetic graphene oxide (mGO) on Reactive Black 5 (RB5) adsorption was investigated. The synthesis of mGO was achieved both with (i) impregnation method (mGOi nanoparticles), and (ii) co-precipitation (mGOp nanoparticles). After synthesis, the full characterization with various techniques (SEM, FTIR, XRD, DTA, DTG, VSM) was achieved revealing many possible interactions/forces of dye-composite system. Effects of initial solution pH, effect of temperature, adsorption isotherms and kinetics were investigated in order to conclude about the aforementioned effect of the preparation method on dye adsorption performance of the magnetic nanocomposites. The adsorption evaluation of the magnetic nanoparticles presented higher adsorption capacity of mGOp derivative (188 mg/g) and lower of mGOi (164 mg/g). Equilibrium experiments are also performed studying the effect of contact time (pseudo-first and -second order equations) and temperature (isotherms at 25, 45 and 65 °C fitted to Langmuir and Freundlich model). A full thermodynamic evaluation was carried out, calculating the parameters of enthalpy, free energy and entropy (ΔH^0^, ΔG^0^ and ΔS^0^).

## 1. Introduction

Among the technologies used for water and wastewater treatment, adsorption proved to be one of the most efficient separation methods for water purification. Adsorption found also to be an effective method treatment for the removal from water and wastewaters of the synthetic dyes that are toxic and cannot be efficiently decolorized by traditional methods. The release of dyes into environment constitutes an important proportion of water pollution [1]. The adsorption of synthetic dyes onto adsorbents is considered as a simple and economical method, especially when applied low-cost adsorbents [2,3,4]. The choice of the appropriate adsorbent materials is very important issue. Carbon materials are well known for their use as excellent adsorbents [5,6].

Graphene, a new class of two dimensional carbon nanostructure with one atom thickness and with a two–dimensional honeycomb sp^2^ carbon lattice, receives extensive research interest due to its unique properties [7] and applications in catalysis, biomedical fields, adsorption and separation *etc.* [8]. The π-electron rich structure renders graphene potential applications as adsorbent [9,10]. Graphene oxide (GO) produced from graphite (G) after chemical oxidation, is one of the most important derivatives of graphene. It is characterized by a layered structure with oxygen functional groups bearing on the basal planes and edges. However, its small particle size and the high dispersibility in aqueous solutions, make difficult its separation from solution after adsorption process via filtration and/or centrifugation. A solution to this problem is the development of magnetic adsorbents, which can ensure the convenient magnetic separation after adsorption.

Magnetic separation has advantages such as its easy phase separation with aqueous solutions and the capability of treating large amount of wastewater in short period of time. Magnetic separation based on the superparamagnetic Fe_3_O_4_ is much more convenient, efficient and economic and have found many interesting applications nowadays. The most important factor for a successful separation is the choice of appropriate magnetic adsorbent materials that dominate the selectivity and sensitivity of the method. Therefore, many works have been published regarding synthesis of magnetic materials and their application for separations [11,12,13,14,15,16], especially for dye removal [6,17] or ions [18]

Many procedures were applied to magnetic graphite oxide fabrication including *in situ* co-precipitation, and covalent bonding [19,20], methods are generally multistep, hard to control and they also require some rigorous conditions. Electrostatic self–assembly has proved to be an effective method for fabricating metal oxides composites especially with carbon based materials [21,22].

In this paper, Fe_3_O_4_/graphene oxide nanocomposites were prepared by impregnating graphene oxide with magnetic nanoparticles (denoted hereafter as mGOi). In order to compare the effect of preparation routes on dye adsorption (Reactive Black 5 used as dye model compound), magnetic nanocomposites were prepared after the co-precipitation method (abbreviated hereafter as mGOp), too. The materials prepared after the two routes were characterized via XRD, TGA, FTIR and SEM. Effects of initial solution pH, effect of temperature, adsorption isotherms and kinetics were investigated in order to conclude about the aforementioned effect of the preparation method on dye adsorption performance of the magnetic nanocomposites.

## 2. Materials and Methods

### 2.1. Materials

FeCl_3_·6H_2_O, FeCl_2_·4H_2_O graphite powder, NH_4_OH, H_2_O_2_ (30% *v*/*v*) and all other reagents were purchased from Sigma-Aldrich and used without further purification. All chemicals used in this study were of analytical grade.

### 2.2. Preparation Routes of Adsorbents

Fe_3_O_4_/graphene oxide nanocomposites were prepared by two different routes: (i) impregnation and (ii) co-precipitation.

#### 2.2.1. Synthesis of Graphene Oxide (GO)

GO was synthesized from natural graphite powder (G) according to the modified Hummers method [23]. In a typical procedure, 120 mL of concentrated H_2_SO_4_ was added into a 500 mL flask containing 5 g of graphite and followed by stirring for 30 min inside an ice bath. Then, 15 g of KMnO_4_ was added slowly to the mixture. The rate of addition was carefully controlled to maintain the reaction temperature below of 20 °C. The mixture was stirred at room temperature overnight. Then, 150 mL of H_2_O was slowly added under vigorous stirring. The reaction temperature rapidly increased to 98 °C, and the color of the mixture was changed into yellow. The diluted suspension was stirred at 98 °C for 24 h. Then, 50 mL of H_2_O_2_ (30% *v*/*v*) was added to the mixture. For purification, the mixture was washed by rinsing and centrifugation with HCl (5% *v*/*v*) followed by deionized water for several times. After filtration and freeze drying, graphite oxide was obtained as a solid.

#### 2.2.2. Synthesis of Fe_3_O_4_ Nanoparticles

Fe_3_O_4_ nanoparticles were prepared according to the modified Massart method [24] via the co-precipitation of a mixture of FeCl_3_·6H_2_O and FeCl_2_·4H_2_O [25]. In particular, FeCl_3_·6H_2_O (3.03 g, 11.2 mmol) and FeCl_2_·4H_2_O (1.13 g, 5.6 mmol) were completely dissolved in 150 mL of deionized water. The aqueous solution was heated to 60 °C so as to obtain a clear yellow solution under vigorous agitation. Then, aqueous ammonia solution was added dropwise until the pH of the solution reached the value of 10. The reaction was maintained for an additional 30 min under vigorous stirring. N_2_ was used as the protective gas throughout the experiment. After the completion of reaction, the black precipitate was collected by an external magnetic field, followed by washing several times with deionized water and ethanol. Finally, the Fe_3_O_4_ nanoparticles were freeze-dried.

#### 2.2.3. Synthesis of Fe_3_O_4_-GO Nanocomposites by co-Precipitation (mGOp)

In a typical synthesis, GO (0.3 g) was dispersed in 150 mL water by sonication for 30 min in order graphene oxide to be formed. Then, 0.825 g FeCl_3_·6H_2_O and 0.322 g of FeCl_2_·4H_2_O were dissolved in 25 mL of water and the solution was added dropwise to GO solution at room temperature under a nitrogen flow with vigorous stirring. After completing ion exchange, 28% ammonia solution was added dropwise to make the pH of solution 10 for synthesis of magnetite nanoparticles. The temperature of the solution rose to 80 °C. After stirring for about 45 min, the black precipitate was centrifuged, washed with ethanol several times, and finally was freeze-dried.

#### 2.2.4. Synthesis of Fe_3_O_4_-GO Nanocomposites by Impregnation (mGOi)

The graphite oxide dispersion (0.3 g GO in 300 mL distilled water) was sonicated for 30 min in order graphene oxide to be formed. An amount of Fe_3_O_4_ nanoparticles (0.3 g) was added to the dispersion. After 30 min of sonication, to obtain a homogenous suspension, the resulted nanocomposites were collected by centrifuging and freeze-dried.

### 2.3. Dye (Reactive Black 5)

**Figure 1:**
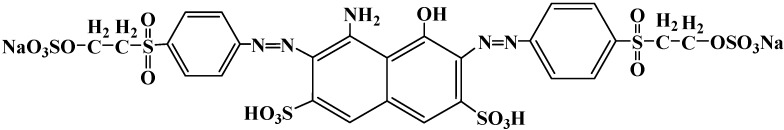
Chemical structures of Reactive Black 5 (RB5).

A commercial reactive dye (anionic and anthraquinonic) was used as target molecule for dye adsorption experiments. The reactive dye, Reactive Black 5—C.I. 20505 (abbreviated hereafter as RB5, supplied by Kahafix), presents the following characteristics: C_26_H_21_N_5_Na_4_O_19_S_6_, MW = 991.82 g/mol, λ_max_ = 603 nm, purity = 55% *w*/*w*. The dye purity was taken into account for all calculations. The chemical structure of the dye used is given in Figure 1.

### 2.4. Characterization

Scanning electron microscopy (SEM) images were performed at Zeiss Supra 55 VP. The accelerating voltage was 15.00 kV and the scanning was performed *in situ* on a sample powder. EDAX analysis was done at magnification 10 K and led to the maps of elements and elemental analysis.

The FTIR spectra of the samples were taken with a FTIR–2000 spectrometer (Perkin Elmer, Dresden, Germany) using KBr disks prepared by mixing 0.5% of finely ground carbon sample in KBr. Pellet made of pure KBr was used as the reference sample for background measurements. The spectra were recorded from 4000 to 400 cm^−1^ at a resolution of 4 cm^−1^. The spectra presented are baseline corrected and converted to the transmittance mode.

Thermal analysis was carried out using a TA Instrument thermal analyzer (SDT) Q500 model (TA Instruments, New York, NY, USA). The instrument had the following settings: (i) heating rate of 10 K/min and (ii) flow rate of nitrogen atmosphere equal to 100 mL/min. Approximately 25 mg of sample was used for each measurement.

X-ray powder diffraction (XRD) patterns were recorded on a PW1820 diffractometer model (Philips, New York, NY, USA) with a CuKα radiation for crystalline phase identification. The sample was scanned from 20° to 80°.

The magnetic property was measured on a vibrating sample magnetometer (VSM) (Oxford Instruments, Oxford, UK) at room temperature.

### 2.5. Adsorption Experiments

The batch adsorption experiments were carried out in 50 mL flasks, where *m* = 0.020 g of the adsorbent and V = 20 mL of the RB5 solutions of different initial concentrations were added in the presence of constant background electrolyte I = 1 M NaCl and pH = 3. The flasks were placed in reciprocating water bath shaker (Julabo SW–21C) under constant temperature environment (*T* = 25 °C) and agitation rate (N = 160 rpm), for 24 h. The initial pH values were adjusted with 0.1 M HCl or 0.1 M NaOH. The effect of initial dye concentration and temperature was determined for *C*_0_ = 0–500 mg/L and *T* = 25, 45, 65 °C. Kinetic experiments were performed at optimum values found (pH = 3) for *C*_0_ = 250 mg/L. The resulted equilibrium data (*C*_e_) were fitted to the Langmuir [26], and Freundlich [27] isotherm models.

Aliquots were taken from the supernatant during the batch adsorption process and the residual dye concentrations of each solution was determined by measuring their absorbance using a double beam UV–Vis spectrophotometer (Hitachi, model U–2000) at 603 nm. The absorbance was converted to concentration using the calibration curve. The equilibrium amount Q_e_ (mg/g) of adsorbed RB5 in the solid phase was calculated using the mass balance equation: Q_e_ = (*C*_0_ − *C*_e_) × (V/m).

Based on the equilibrium data of isotherms, thermodynamic parameters were calculated. The Gibbs free energy change, ΔG^0^ (kJ/mol), of the adsorption process is related to the equilibrium constant (K_c_) by the Van’t Hoff equation (where R is the universal gas constant and is equal to 8.314 J/mol K) [28]:

(1) $$\text{Δ}G^{0}=-\text{R T ln}(K_{c})$$

The constant K_c_ can be calculated as *K*_c_ = *C*_s_/*C*_e_ (where *C*_s_ (mg/L) is the amount adsorbed on solid at equilibrium). In addition, ΔG^0^ is related to the change in entropy (ΔS^0^, kJ/mol K) and the heat of adsorption (ΔH^0^, kJ/mol) at a constant temperature *T* (K), as follows:

(2) $$\text{Δ}G^{0}=\text{Δ}H^{0}-\text{T Δ}S^{0}$$

From Equations (1) and (2):

(3) $$\text{ln}(K_{c})=(\frac{-\text{Δ}H^{0}}{R})\frac{1}{T}+\frac{\text{Δ}S^{0}}{R}$$

The values of ΔH^0^ and ΔS^0^ were calculated from the slope and intercept of the plot between ln(*K*_c_) *versus* (1/*T*).

## 3. Results and Discussion

### 3.1. Characterization

**Figure 2:**
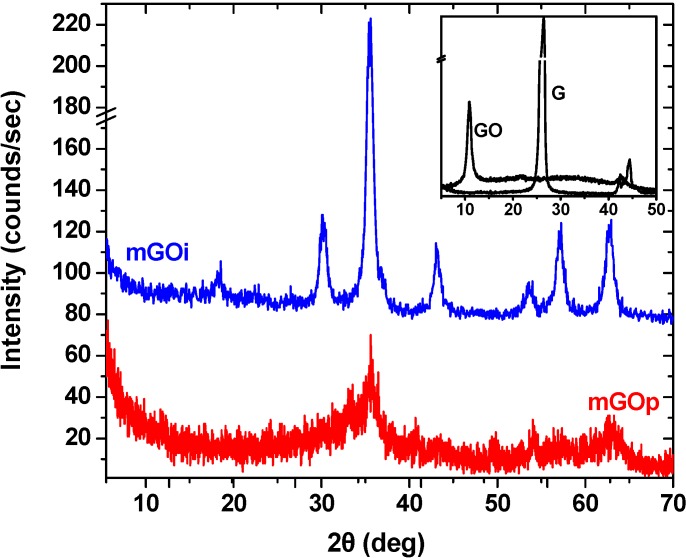
X-ray diffraction (XRD) patterns of mGOi and mGOp (Inset: XRD patterns of GO and G).

X-ray diffraction measurements (XRD) were performed in order to obtain crystalline structural information for the magnetic composites prepared after the two preparation routes (impregnation and co-precipitation). Figure 2 presents the X-ray diffraction (XRD) measurements of mGOi and mGOp nanocomposites. In the inset, the XRD pattern of the parent graphite and the graphite oxide are presented. The sharp d_002_ peak of the graphite at 2θ = 26.1° indicates an interlayer spacing of 0.336 nm and after oxidation, this characteristic graphite peak disappeared and replaced by a well defined peak at 2θ = 10.9° with 0.81 nm d–spacing. This increased d-spacing of GO sheets is due to the presence of abundant oxygen containing functional groups on both sites of the graphene sheet.

For the mGOi, the position and relative intensities of all diffraction peaks at 2θ values of 18.27° (111), 30.1° (220), 35.4° (311), 43.05° (400), 56.94° (422), 62.51° (511) and 73.95° (553) are consistent with the standard XRD data for the spinel structure Fe_3_O_4_ with lattice constants of α = 8.397 Å (indexed using Joint Committee on Power Diffraction Standards database (JCPDS 19–0629)) [29].

The average crystallite size D (nm) of the Fe_3_O_4_ particles is calculated using the Debye-Sherrer equation [21,30]:

(4) $$D=\frac{K_{s}\cdot \text{λ}}{B\cdot \text{cos θ}}$$

where K_s_ is a constant (*K*_s_ = 0.9 for CuKa), λ (nm) is wavelength (0.15405 nm for CuKa), B is the peak width of half–maximum (rad) and θ is the diffraction angle.

The average size of the Fe_3_O_4_ particles using 35.56° diffraction peak was found to be ~18.4 nm. The (001) diffraction peak of GO at 10.3° (inset of Figure 2) totally disappeared, suggesting that the layered GO has been exfoliated in the preparation process of mGOi nanocomposite. The absence of the peak at 2θ = 10.3°, suggests the complete exfoliation of graphite oxide during the preparation process, while a broad small peak at 2θ = 24.4° (002) corresponding to the graphene sheets indicated the formation of the Fe_3_O_4_/GO nanocomposites.

The XRD pattern of the mGOp is different. It appeared to be amorphous, may be due to the effect of the ammonia solution. However wide peaks are observed at 2θ about at 30.1° and ~57.2° that can be attributed to (220) and (511) of Fe_3_O_4_ and a small peak at 2θ = 24.4° (002) corresponding to the graphene sheets, indicating this way the formation of the magnetic composite.

**Figure 3:**
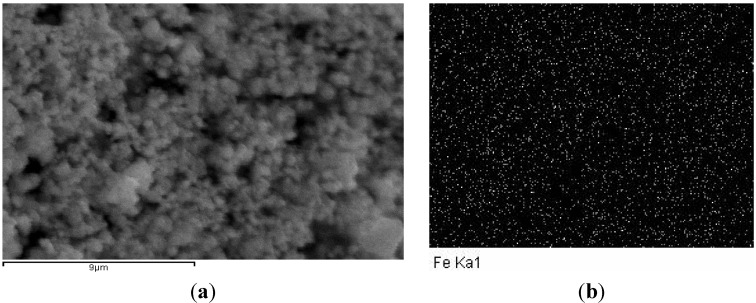
(**a**) SEM image of mGOi; (**b**) iron distribution map of mGOi.

A SEM image of the mGOi nanoparticles is presented in Figure 3a, according to which the nano-size of particles prepared are evident. Furthermore, Figure 3b presents the iron distribution map of mGOi, which indicates that Fe is well distributed in the prepared magnetic composite.

**Figure 4:**
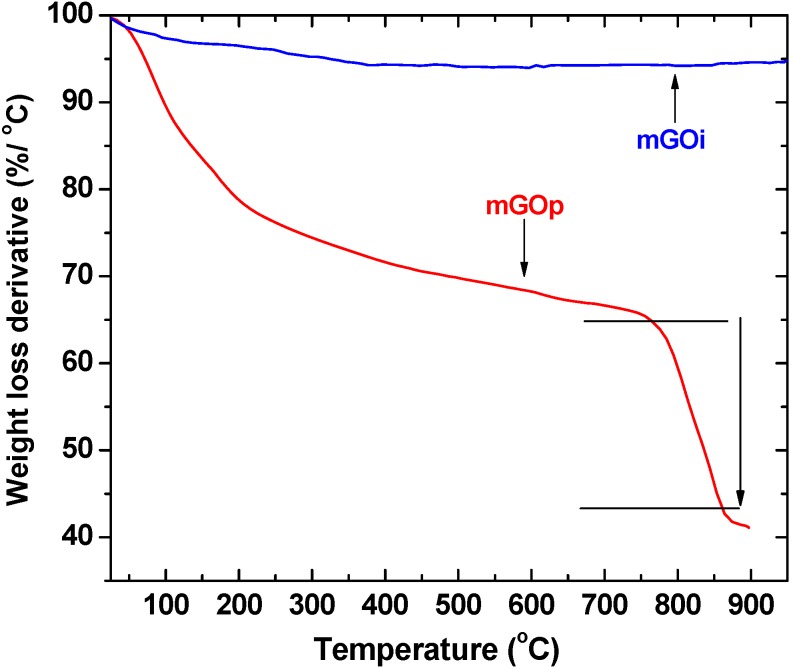
Differential thermogravimetry (DTG) curves in nitrogen.

Differences in the chemistry of the surfaces for the prepared nanocomposites have been also seen on the differential thermal gravimetric (DTG) curve measured in nitrogen (Figure 4), where the peaks represent weight loss at the specific temperature range and the area under the peaks is related to the extent of that weight loss, is presented for mGOp sample. The DTG curve of graphene oxide is also presented for comparison.

**Figure 5:**
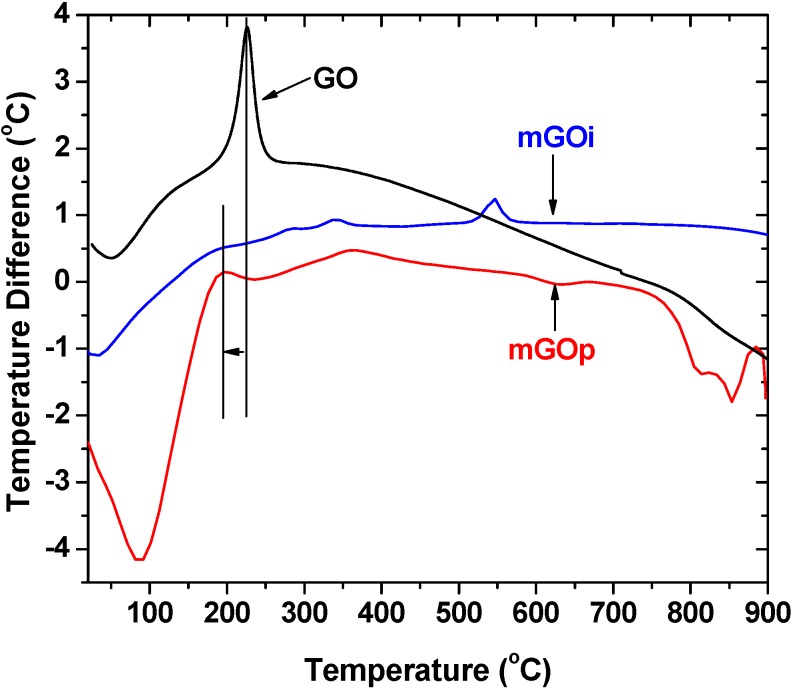
Differential thermal analysis (DTA) curves in nitrogen.

The first peak centered at about 80–100 °C for all under examination samples, in correlation to the endothermic effect on the differential thermal analysis (DTA) curves presented in Figure 5 can be linked to weight loss due to the evaporation of physically adsorbed water.

In the case of the mGOp composite, that peak presented a maxima at 100 °C. The exothermic peak, from 200 to 250 °C as presented on the differential thermal analysis (DTA) curve in Figure 5 for the GO sample, is related to decomposition of epoxy and carboxyl groups. This peak is no longer observed for the mGOi nanocomposite since the layered GO has been exfoliated in the preparation process of graphene oxide and mGOi nanocomposite, while only a small peak is presented for the mGOs sample may be due to some small residue of GO.

For this sample a significant part of the weight loss at temperatures above 750 °C must be related to the gradual dehydratation/dehydroxylation of iron oxyhydroxides [31]. Their formation during the co-precipitation preparation route has been reported [32,33,34,35] and their existence is not testified in the XRD may be due to the amorphous appearance of the XRD pattern of this magnetic material. These oxyhydroxides are finally reduced in two steps to metallic iron between 750 and 900 °C [31] and from the weight loss curve it is concluded that their percentage is about 22%. No weight loss was occurred for the mGOi samples after 530 °C.

**Figure 6:**
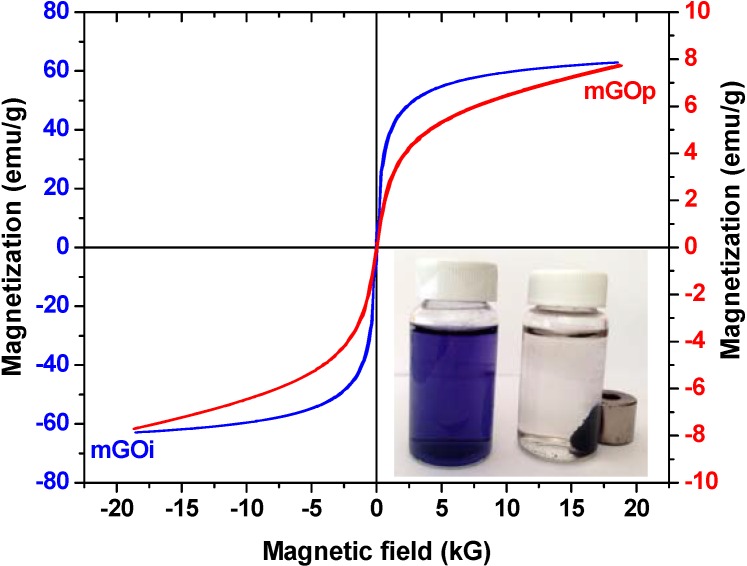
VSM plot of mGOi and mGOp (Inset: photo of mGOp).

The magnetization hysteresis loop of mGOi and mGOp nanocomposites (at room temperature) is shown in Figure 6. The magnetization curves were S-like curves and presented zero coercivity and permanence indicating this way the superparamagnetic property of the nanocomposite. The saturation magnetization of the mGOi magnetic adsorbent found to be about 65 emu/g, while the respective value of mGOp was found to be considerable smaller. This can be attributed to the smaller percentage of magnetite in this sample due to the formation of iron oxyhydroxides, as seen by DTA measurements. In both cases, the saturation magnetization was strong enough for a convenient magnetic separation.

Inset of Figure 6 presents a photograph of an aqueous RB5 solution The left flask is the aqueous solution of RB5 (before adsorption), while the right flask illustrated the same solution after adsorption of RB5 onto mGOp. In the presence of an external magnetic field, the black particles of the magnetic composite (mGOp) were attracted to the wall of vial in order to emphasize the magnetism of mGOp prepared.

**Figure 7:**
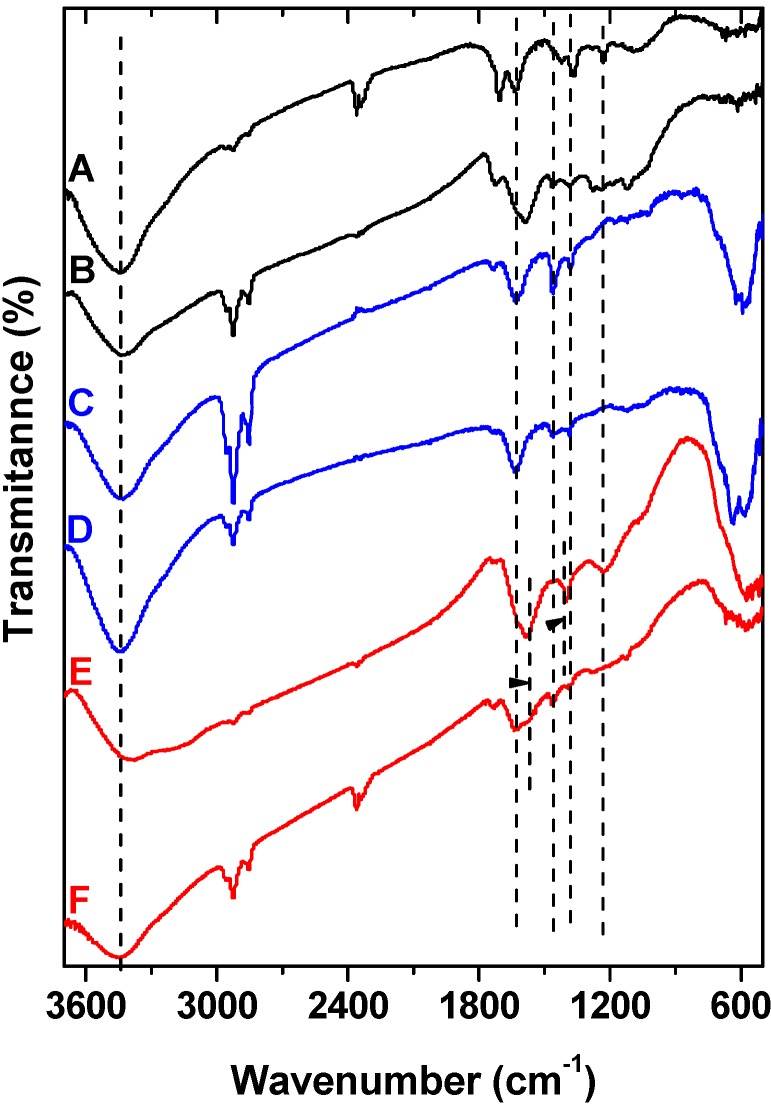
FT–IR spectra of: (**A**) GO; (**B**) GO dye–loaded; (**C**) mGOi; (**D**) mGOi dye-loaded; (**E**) mGOp; (**F**) mGOp dye–loaded.

FTIR of the parent graphite oxide as well as of the prepared nanocomposites are presented in Figure 7. All the samples present the O–H stretching vibration adsorption band at 3424 cm^−1^ and the band at ~1600 cm^−1^, which is attributed to C=C stretching mode of the sp^2^ carbon skeletal network. Carboxylic groups of GO (spectrum A) are observed as bands at 1720 cm^−1^ and 1380 cm^−1^ as the stretching vibration peaks of carboxyl and carbonyl while the bands at 1055 cm^−1^ and 1230 cm^−1^ can be attributed to the stretching vibrations of C–O of epoxy groups. The C=O groups would facilitate the attachment of magnetic nanoparticles through a covalent coupling or electrostatic interaction [36].

The spectra of mGOi (C) and mGOp (E) nanocomposites additionally present the characteristic stretching vibration peak at 568 cm^−1^ that proved that Fe_3_O_4_ nanoparticles was successfully anchored onto their graphene sheets. These materials also presented the adsorption peaks related to oxygen containing functional groups, but at a decreased intensity may be due to the formation of –COO^−^ after coating with Fe_3_O_4_ [37]. Therefore, it is concluded that Fe_3_O_4_ nanoparticles are chemically deposited on GO with the aid of the –COOH on graphene oxide. The mGOp nanocomposite presented an additional band at 1400 cm^−1^, which represents the vibrations of nitrogen in NH_4_^+^ inorganic ion. NH_4_^+^ is a Brönsted acid, which can react with deprotonated carboxylic type acidic sites and ammonium salts are formed, or get adsorbed as NH_4_^+^ on the metal acidic sites [38]. On the other hand the presence of iron, favors Lewis acid–base interactions, offering/contributing in this way binding sites for ammonium ions [38]. This band appeared no more after the dye adsorption due to reaction of these ions with the sulfonate groups of the RB5. In addition, Figure 7 shows the spectra of adsorbents after dye adsorption (spectra B, D, F). In the case of GO, the shift of the band (spectrum B) is indicative that the binding of dye molecules is predominant based on the π-π interaction between the aromatic ring of the dye and the GO basal planes [39].

### 3.2. Adsorption Experiments

Firstly, pH–effect tests were carried out in order to find the optimum pH value. As it was found, the optimum value was 3 for both magnetic GO–based materials (data not shown). The same pH–behavior was also found in many works in literature [39], which is directly related with the nature of interactions between RB5 molecules and GO layers. The latter is mainly attributed to the π-π dispersion interaction between the aromatic ring of the dye (see Figure 1) and the GO basal planes [39].

#### 3.2.1. Kinetics

**Figure 8:**
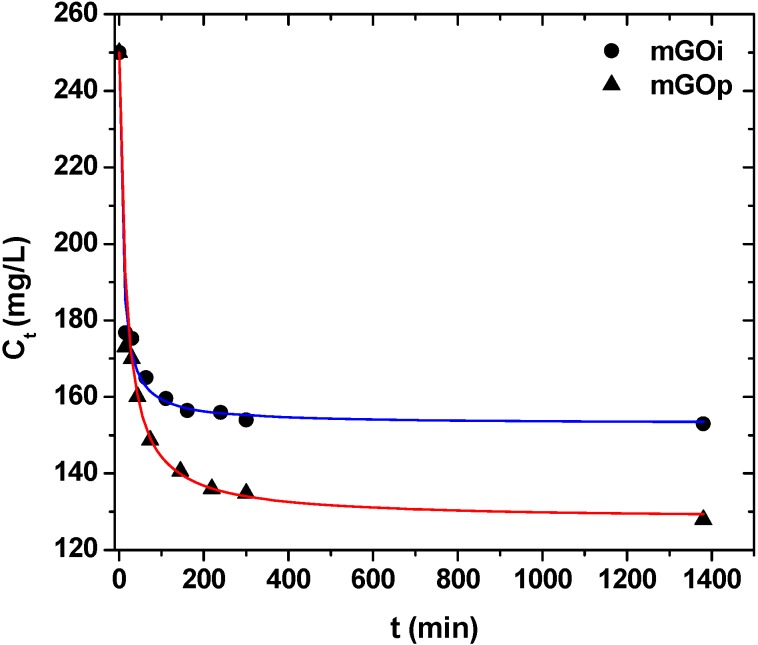
Effect of contact time on RB5 removal.

Figure 8 illustrates the effect of contact time on dye removal with mGOi and mGOp adsorbents. The plots could be divided in three zones: (i) 0–30 min, which indicated the instantaneous adsorption of dyes, suggesting rapid external diffusion and surface adsorption; (ii) 30–120 min, showed a gradual equilibrium, and (iii) 2–24 h, indicated the plateau of the equilibrium state [40]. It can be seen that the adsorption was rapid at the initial stage of the contact, but it gradually slowed down until the equilibrium. The fast adsorption at the initial stage can be attributed to the fact that a large number of surface sites are available for adsorption. After a lapse of time, the remaining surface sites are difficult to be occupied because of the repulsion between the solute molecules of the solid and bulk phases make it took long time to reach equilibrium [41,42]. Table 1 presents the kinetic parameters resulted from the fitting of the pseudo–first and –second order equations to the experimental kinetic data. Based on the correlation coefficients (R^2^) exported, the best fitting (for both adsorbents tested) was observed for the pseudo-second order equation (R^2^ > 0.990), while the pseudo–first order model presented enough lower coefficients (R^2^ < 0.954). The kinetic rate was established with the parameter “k” calculated, as shown in Table 1.

**Table 1:** Kinetic constants for RB5 removal with mGOi and mGOp at 25 °C.

Adsorbent	Pseudo–first order		Pseudo–second order	
	k_1_ (min^−1^)	R^2^	k_2_ (min^−1^)	R^2^
mGOi	0.072	0.915	0.143	0.990
mGOp	0.029	0.954	0.064	0.991

#### 3.2.2. Effect of Initial Dye Concentration and Temperature (Isotherms)

The experimental data were fitted to the Langmuir [26], and Freundlich [27] isotherm model. Although the Langmuir and Freundlich isotherms were firstly introduced about 90 years ago, they still remain the two most commonly used adsorption isotherm equations. Their success undoubtedly reflects their ability to fit a wide variety of sorption data quite well. The Langmuir model represents chemisorption on a set of well defined localized adsorption sites, having the same adsorption energies independent of surface coverage and no interaction between adsorbed molecules. Langmuir isotherm assumes monolayer coverage of adsorbate onto adsorbent. Freundlich isotherm gives an expression encompassing the surface heterogeneity and the exponential distribution of active sites and their energies. This isotherm does not predict any saturation of the adsorbent surface; thus, infinite surface coverage is predicted, indicating physisorption on the surface.

**Figure 9:**
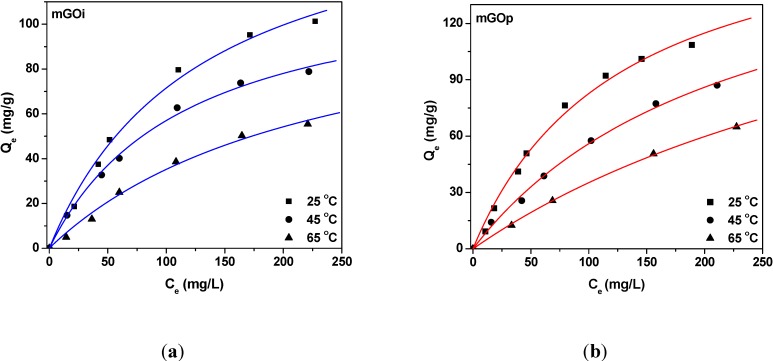
Isotherms of the adsorption of RB5 onto (**a**) mGOi and (**b**) mGOp.

Figure 9a,b presents the isotherms resulted from the adsorption of RB5 onto magnetic graphene oxide materials. Furthermore, Table 2 reports the maximum adsorption capacities (Q_max_) and the other isothermal parameters resulted from the fitting. The correlation coefficients (R^2^ > 0.989), which is an indication of the successful fitting, confirm that the Langmuir model results in closer prediction of the isotherm to the experimental data. The calculated maximum adsorption capacities (Q_max_) for RB5 removal at 25 °C (pH = 3) was 164 and 188 mg/g for mGOi, and mGOp, respectively.

**Table 2:** Equilibrium parameters for the adsorption of RB5 onto mGOi and mGOp at 25, 45 and 65 °C.

Adsorbents	Langmuir equation				Freundlich equation		
	T (°C)	Q_max_ (mg/g)	K_L_ (L/mg)	R^2^	K_F_ (mg^(n−1)/n^ L^1/n^ g^−1^)	*n*	R^2^
mGOi	25	164	0.007	0.989	4.55	0.58	0.960
	45	124	0.008	0.993	4.13	0.55	0.970
	65	118	0.004	0.990	1.24	0.71	0.973
mGOp	25	188	0.007	0.991	4.24	0.63	0.966
	45	186	0.004	0.995	2.17	0.69	0.988
	65	178	0.002	0.999	0.84	0.80	0.995

For all the adsorbents studied the equilibrium dye uptake was affected by the initial dye concentration using constant dosage of adsorbent (0.020 g per 20 mL). At low initial concentrations, the adsorption of dye is very intense and reaches equilibrium rapidly. This phenomenon indicates the possibility of the formation of monolayer coverage of dye molecules at the outer interface of GO-based materials. Furthermore, for low concentrations (0–50 mg/L) the ratio of initial number of dye molecules to the available adsorption sites is low and subsequently the fractional adsorption becomes independent on initial concentration [43]. Brunauer *et al.* divided the isotherms of adsorption into five types [44]. Type I isotherms represents unimolecular adsorption and applies to non-porous, microporous and adsorbents with small pore sizes (not significantly greater than the molecular diameter of the adsorbate). So, the shapes of curves (Figure 9) indicate that the isotherms for the adsorbent–dye systems studied are I–Type, according to the BET classification [44], and characterized by a high degree of adsorption at low concentrations. At higher concentrations, the available adsorption sites become lower and subsequently the adsorption depends on the initial concentration of dye. As a matter of fact, the diffusion of exchanging molecules within GO particles may govern the adsorption rate at higher initial concentrations. The effect of temperature on equilibrium is presented through isotherms curves (Figure 9a,b). Increasing the temperature of process from 25 to 65 °C, a decrease of the adsorption capacity (dye uptake) was observed.

### 3.3. Thermodynamics

The parameters of ΔH^0^ and ΔS^0^ were calculated from the slope and intercept of the plot between ln(K_c_) *versus* (1/T) (R^2^ > 0.985, data not shown). These parameters, at selected dye concentrations and all temperatures, are given in Table 3 (pH = 3). The negative values of ΔG^0^ showed the spontaneous adsorption of reactive dyes on the adsorbent. In addition, the increase found in the negative value of ΔG^0^ with an increase in temperature implies that lower temperature makes the adsorption easier. Negative value of ΔH^0^ indicates the exothermic nature of the process, thereby demonstrating that the process is stable energetically. In an exothermic process, the total energy absorbed in bond breaking is less than the total energy released in bond making between adsorbate and adsorbent, resulting in the release of extra energy in the form of heat. Therefore, ΔH^0^ will be negative. The magnitude of ΔH^0^ may also give an idea (but not sure) about the type of adsorption. The heat evolved during physical adsorption is of the same order of magnitude as the heats of condensation, *i.e.*, 2.1–20.9 kJ/mol, while the heats of chemisorption generally falls into a range of 80–200 kJ/mol. Moreover, the values of ΔS^0^ were found to be negative. The latter suggests that the adsorption process is enthalpy driven. In general, a negative value of entropy change (ΔS^0^) also implies a decreased disorder at the solid/liquid interface during the adsorption process causing the adsorbate ions/molecules to escape from the solid phase to the liquid phase. Therefore, the amount of adsorbate that can be adsorbed will decrease. During the adsorption, the coordinated water molecules (which are displaced by the dye molecules) gain more translational entropy than is lost by the dye molecules, resulting in increased randomness in the dye–adsorbent interaction [45,46]. It is well known that ionic dyes trend to aggregate in dilute solutions, leading to dimmer formation [47,48]. It is supposed that dimmer formation in solution is mainly due to hydrophobic interactions or permanent and transition dipole moments [47,48]. Although dyes are very individualistic in structure, certain broad rules are well established regarding their dimerization. The probability increases with an increase of dye concentration or ionic strength; it will decrease with temperature rising or organic solvents adding [47,48]. In the current study, the use of a commercial reactive dye, which is not pure, prevents from an obvious conclusion in this topic.

**Table 3:** Thermodynamic parameters for the adsorption of RB5 onto mGOi and mGOp.

Adsorbent	*C*_0_ (mg/L)	*T* (K)	Q_e_ (mg/g)	*K*_c_	ΔG^0^ (kJ/mol)	ΔH^0^ (kJ/mol)	ΔS^0^ (kJ/mol K)
mGOi	40	298	18.02	0.82	-0.50	-24.83	-0.085
		318	12.01	0.43	-2.24		
		338	8.09	0.25	-3.90		
	100	298	48.56	0.92	-0.20	-23.54	-0.071
		318	40.75	0.67	-1.07		
		338	25.41	0.33	-3.09		
	300	298	130.02	0.76	-0.66	-21.13	-0.082
		318	79.99	0.36	-2.67		
		338	60.01	0.25	-3.90		
mGOp	20	298	22.02	1.22	-0.50	-27.04	-0.089
		318	17.09	0.74	-0.80		
		338	10.08	0.33	-3.09		
	80	298	52.10	1.08	-0.20	-20.32	-0.067
		318	42.12	0.72	-0.85		
		338	29.03	0.41	-2.52		
	300	298	145.62	0.94	-0.17	-19.31	-0.068
		318	95.58	0.46	-2.03		
		338	81.04	0.37	-2.80		

## 4. Conclusions

In this study, the effect of preparation route of magnetic graphene oxide (mGO) on Reactive Black 5 (RB5) adsorption was investigated. The synthesis of mGO was achieved both with (i) impregnation method (mGOi nanoparticles), and (ii) co-precipitation (mGOp nanoparticles).

The average size of the Fe_3_O_4_ particles using 35.56° diffraction peak was found to be ~18.4 nm. The (001) diffraction peak of GO at 10.3° totally disappeared, suggesting that the layered GO has been exfoliated in the preparation process of mGOi nanocomposite. The absence of the peak at 2θ = 10.3°, suggests the complete exfoliation of graphite oxide during the preparation process, while a broad small peak at 2θ = 24.4° (002) corresponding to the graphene sheets indicated the formation of the Fe_3_O_4_/GO nanocomposites. The XRD pattern of the mGOp is different. It appeared to be amorphous, may be due to the effect of the ammonia solution. In addition, the saturation magnetization of the mGOi magnetic adsorbent found to be approximately 65 emu/g, while the respective value of mGOp was found to be considerable smaller. For all the adsorbents studied the equilibrium dye uptake was affected by the initial dye concentration using constant dosage of adsorbent (0.020 g per 20 mL). At low initial concentrations, the adsorption of dye is very intense and reaches equilibrium rapidly. This phenomenon indicates the possibility of the formation of monolayer coverage of dye molecules at the outer interface of GO-based materials. Based on the correlation coefficients (R^2^) exported, the best fitting (for both adsorbents tested) was observed for the pseudo-second order equation (R^2^ > 0.990), while the pseudo–first order model presented enough lower coefficients (R^2^ < 0.954). From a thermodynamic point of view, the values of ΔS^0^ were found to be negative. The latter suggests that the adsorption process is enthalpy driven. The negative values of ΔG^0^ showed the spontaneous adsorption of reactive dyes on the adsorbent. In addition, the increase found in the negative value of ΔG^0^ with an increase in temperature implies that lower temperature makes the adsorption easier. Negative value of ΔH^0^ indicates the exothermic nature of the process, thereby demonstrating that the process is stable energetically.

## References

[1] Forgacs E., Cserháti T., Oros G. (2004). Removal of synthetic dyes from wastewaters: A review. Environ. Int..

[2] Kyzas G.Z. (2012). A decolorization technique with spent “Greek coffee” grounds as zero-cost adsorbents for industrial textile wastewaters. Materials.

[3] Kyzas G.Z. (2012). Commercial coffee wastes as materials for adsorption of heavy metals from aqueous solutions. Materials.

[4] Kyzas G.Z., Lazaridis N.K., Mitropoulos A.C. (2012). Removal of dyes from aqueous solutions with untreated coffee residues as potential low-cost adsorbents: Equilibrium, reuse and thermodynamic approach. Chem. Eng. J..

[5] Mezohegyi G., van der Zee F.P., Font J., Fortuny A., Fabregat A. (2012). Towards advanced aqueous dye removal processes: A short review on the versatile role of activated carbon. J. Environ. Manage..

[6] Tiwari J.N., Mahesh K., Le N.H., Kemp K.C., Timilsina R., Tiwari R.N., Kim K.S. (2013). Reduced graphene oxide-based hydrogels for the efficient capture of dye pollutants from aqueous solutions. Carbon.

[7] Yan J., Wei T., Qiao W., Shao B., Zhao Q., Zhang L., Fan Z. (2010). Rapid microwave-assisted synthesis of graphene nanosheet/Co_3_O_4_ composite for supercapacitors. Electrochim. Acta.

[8] Zhang Y., Chen B., Zhang L., Huang J., Chen F., Yang Z., Yao J., Zhang Z. (2011). Controlled assembly of Fe_3_O_4_ magnetic nanoparticles on graphene oxide. Nanoscale.

[9] Zhuo Y., Yuan P.X., Yuan R., Chai Y.Q., Hong C.L. (2009). Bienzyme functionalized three-layer composite magnetic nanoparticles for electrochemical immunosensors. Biomaterials.

[10] Yin H., Zhou Y., Ma Q., Ai S., Chen Q., Zhu L. (2010). Electrocatalytic oxidation behavior of guanosine at graphene, chitosan and Fe_3_O_4_ nanoparticles modified glassy carbon electrode and its determination. Talanta.

[11] Zhu J., Pallavkar S., Chen M., Yerra N., Luo Z., Colorado H.A., Lin H., Haldolaarachchige N., Khasanov A., Ho T.C. (2013). Magnetic carbon nanostructures: Microwave energy-assisted pyrolysis *vs.* conventional pyrolysis. Chem. Commun..

[12] Zhua J., Sadua R., Weib S., Chena D., Haldolaarachchige N., Luo Z., Gomesa J.A., Young D.P., Guo Z. (2012). Magnetic graphene nanoplatelet composites toward arsenic removal. ECS J. Solid State Sci. Technol..

[13] Zhu J., Wei S., Gu H., Rapole S.B., Wang Q., Luo Z., Haldolaarachchige N., Young D.P., Guo Z. (2012). One-pot synthesis of magnetic graphene nanocomposites decorated with core@double-shell nanoparticles for fast chromium removal. Environ. Sci. Technol..

[14] Zhang D., Wei S., Kaila C., Su X., Wu J., Karki A.B., Young D.P., Guo Z. (2010). Carbon-stabilized iron nanoparticles for environmental remediation. Nanoscale.

[15] Gu H., Rapole S.B., Sharma J., Huang Y., Cao D., Colorado H.A., Luo Z., Haldolaarachchige N., Young D.P., Walters B. (2012). Magnetic polyaniline nanocomposites toward toxic hexavalent chromium removal. RSC Adv..

[16] Zhu J., Gu H., Rapole S.B., Luo Z., Pallavkar S., Haldolaarachchige N., Benson T.J., Ho T.C., Hopper J., Young D.P. (2012). Looped carbon capturing and environmental remediation: Case study of magnetic polypropylene nanocomposites. RSC Adv..

[17] Travlou N.A., Kyzas G.Z., Lazaridis N.K., Deliyanni E.A. (2013). Functionalization of graphite oxide with magnetic chitosan for the preparation of a nanocomposite dye adsorbent. Langmuir.

[18] Chandra V., Park J., Chun Y., Lee J.W., Hwang I.C., Kim K.S. (2010). Water-dispersible magnetite-reduced graphene oxide composites for arsenic removal. ACS Nano.

[19] He F., Fan J., Ma D., Zhang L., Leung C., Chan H.L. (2010). The attachment of Fe_3_O_4_ nanoparticles to graphene oxide by covalent bonding. Carbon.

[20] Georgakilas V., Otyepka M., Bourlinos A.B., Chandra V., Kim N., Kemp K.C., Hobza P., Zboril R., Kim K.S. (2012). Functionalization of graphene: Covalent and non-covalent approaches, derivatives and applications. Chem. Rev..

[21] Yao Y., Miao S., Yu S., Ping Ma L., Sun H., Wang S. (2012). Fabrication of Fe_3_O_4_/SiO_2_ core/shell nanoparticles attached to graphene oxide and its use as an adsorbent. J. Colloid Interface Sci..

[22] Kim K.S., Zhao Y., Jang H., Lee S.Y., Kim J.M., Kim K.S., Ahn J.-H., Kim P., Choi J.-Y., Hong B.H. (2009). Large-scale pattern growth of graphene films for stretchable transparent electrodes. Nature.

[23] Hummers W.S., Offeman R.E. (1958). Preparation of graphitic oxide. J. Am. Chem. Soc..

[24] Massart R. (1981). Preparation of aqueous magnetic liquids in alkaline and acidic media. IEEE Trans. Magn..

[25] Han Q., Wang Z., Xia J., Chen S., Zhang X., Ding M. (2012). Facile and tunable fabrication of Fe_3_O_4_/graphene oxide nanocomposites and their application in the magnetic solid-phase extraction of polycyclic aromatic hydrocarbons from environmental water samples. Talanta.

[26] Langmuir I. (1918). The adsorption of gases on plane surfaces of glass, mica and platinum. J. Am. Chem. Soc..

[27] Freundlich H. (1906). Over the adsorption in solution. Z. Phys. Chem..

[28] Smith J.M., van Ness H.C. (1987). Introduction to Chemical Engineering Thermodynamics.

[B29-materials-06-01360] 29.Joint Committee on Power Diffraction Standards in International Centre for Diffraction Data: Newtown Square, PA, USA, 2005;Card No. 19–0629

[30] Seung H.H., Mikhailov S. (2011). Thermal Reduction of Graphene Oxide. Physics and Applications of Graphene—Experiments.

[31] Deliyanni E., Bandosz T.J. (2011). Importance of carbon surface chemistry in development of iron-carbon composite adsorbents for arsenate removal. J. Hazard. Mater..

[32] Kanel S.R., Greneche J.M., Choi H. (2006). Arsenic(V) removal from groundwater using nano scale zero-valent iron as a colloidal reactive barrier material. Environ. Sci. Technol..

[33] Kanel S.R., Manning B., Charlet L., Choi H. (2005). Removal of arsenic(III) from groundwater by nanoscale zero-valent iron. Environ. Sci. Technol..

[34] Carpenter E.E., Calvin S., Stroud R.M., Harris V.G. (2003). Passivated iron as core-shell nanoparticles. Chem. Mater..

[35] Fan M., Yuan P., Zhu J., Chen T., Yuan A., He H., Chen K., Liu D. (2009). Core-shell structured iron nanoparticles well dispersed on montmorillonite. J. Magn. Magn. Mater..

[36] Rattana, Chaiyakun S., Witit-anun N., Nuntawong N., Chindaudom P., Oaew S., Kedkeaw C., Limsuwan P. (2012). Preparation and characterization of graphene oxide nanosheets. Procedia Eng..

[37] Bai L.-Z., Zhao D.-L., Xu Y., Zhang J.-M., Gao Y.-L., Zhao L.-Y., Tang J.-T. (2012). Inductive heating property of graphene oxide–Fe_3_O_4_ nanoparticles hybrid in an AC magnetic field for localized hyperthermia. Mater. Lett..

[38] Huang C.C., Li H.S., Chen C.H. (2008). Effect of surface acidic oxides of activated carbon on adsorption of ammonia. J. Hazard. Mater..

[39] Travlou N.A., Kyzas G.Z., Lazaridis N.K., Deliyanni E.A. (2013). Graphite oxide/chitosan composite for reactive dye removal. Chem. Eng. J..

[40] Wang L., Wang A. (2008). Adsorption properties of congo red from aqueous solution onto *N*,*O*-carboxymethyl-chitosan. Bioresour. Technol..

[41] Crini G. (2008). Kinetic and equilibrium studies on the removal of cationic dyes from aqueous solution by adsorption onto a cyclodextrin polymer. Dyes Pigm..

[42] Ho Y.S., McKay G. (1999). Pseudo-second order model for sorption processes. Process Biochem..

[43] Chatterjee S., Chatterjee B.P., Das A.R., Guha A.K. (2005). Adsorption of a model anionic dye, eosin Y, from aqueous solution by chitosan hydrobeads. J. Colloid Interface Sci..

[44] Brunauer S., Deming L.S., Deming W.E., Teller E. (1940). On a theory of the van der Waals adsorption of gases. J. Am. Chem. Soc..

[45] Kyzas G.Z., Kostoglou M., Lazaridis N.K. (2009). Copper and chromium(VI) removal by chitosan derivatives-Equilibrium and kinetic studies. Chem. Eng. J..

[46] Unnithan M.R., Anirudhan T.S. (2001). The kinetics and thermodynamics of sorption of chromium(VI) onto the iron(III) complex of a carboxylated polyacrylamide-grafted sawdust. Ind. Eng. Chem. Res..

[47] Kyzas G.Z., Kostoglou M., Vassiliou A.A., Lazaridis N.K. (2011). Treatment of real effluents from dyeing reactor: Experimental and modeling approach by adsorption onto chitosan. Chem. Eng. J..

[48] Niazi A., Yazdanipour A., Ghasemi J., Kubista M. (2006). Spectrophotometric and thermodynamic study on the dimerization equilibrium of ionic dyes in water by chemometrics method. Spectrochim. Acta A.

